# Functional Outcome of Ultrasound-Guided Caudal Epidural Triamcinolone Acetonide Injection in Patients With Prolapsed Lumbar Intervertebral Disc (PLID) With Radiculopathy

**DOI:** 10.7759/cureus.105180

**Published:** 2026-03-13

**Authors:** Taposh K Ghosh, Sunam K Barua, Suriya Shahaly, Shohel Ahmed, Rayhan Sharif, Md. Sakib A Nahian

**Affiliations:** 1 Physical Medicine and Rehabilitation, National Institute of Traumatology and Orthopedic Rehabilitation (NITOR), Dhaka, BGD; 2 Physical Medicine and Rehabilitation, Ahsania Mission Cancer and General Hospital, Dhaka, BGD; 3 Physical Medicine and Rehabilitation, Eastern Medical College and Hospital, Cumilla, BGD; 4 Physical Medicine and Rehabilitation, Green Life Medical College, Dhaka, BGD

**Keywords:** corticosteroids, oswestry disability index (odi) scale, prolapsed lumbar intervertebral disc (plid), ultrasound-guided caudal epidural injection, visual analog scale (vas)

## Abstract

Introduction

Prolapsed lumbar intervertebral disc (PLID) with radiculopathy is a prevalent cause of low back pain, often treated conservatively or with invasive interventions such as epidural steroid injections (ESIs). Ultrasound-guided caudal ESIs have gained attention due to their accuracy and reduced complication rates compared to traditional methods. This study aimed to assess the efficacy of ultrasound-guided caudal epidural triamcinolone acetonide injection in managing PLID with radiculopathy, in comparison to physical therapy (PT).

Methods

This quasi-experimental study was conducted at Dhaka Medical College Hospital between May 1, 2022 and March 31, 2023. Seventy-one patients with clinically diagnosed and MRI-confirmed PLID were non-randomly allocated into two groups: Group A (n=35) received ultrasound-guided caudal epidural triamcinolone acetonide injections, while Group B (n=36) underwent PT. Pain and functional disability were assessed at baseline and after four, six, and 12 weeks, using the visual analogue scale (VAS) and the Oswestry disability index (ODI). During follow-up, one patient from each group was lost at weeks 1 and 4, respectively. Additionally, three patients from each group dropped out by week 12, resulting in 64 patients (30 from Group A and 34 from Group B) for analysis. The missing data from these dropouts were excluded from the final statistical analysis. Statistical analysis was conducted using IBM SPSS Statistics for Windows, Version 26 (Released 2018; IBM Corp., Armonk, New York, United States). Means and standard deviations were used for continuous variables, while categorical variables were analyzed with chi-square tests, and group differences were assessed using independent and paired t-tests, with p<0.05 considered significant.

Results

At baseline, the mean VAS scores were comparable between Group A and Group B (7.8 vs. 7.4; p = 0.071). By the fourth week, VAS scores had significantly decreased in both groups, with a greater reduction observed in Group A compared to Group B (2.6 vs. 3.6; p = 0.004). At the sixth and 12th weeks, Group A continued to demonstrate significantly lower VAS scores than Group B (p < 0.001). Similarly, baseline ODI scores did not differ significantly between Group A and Group B (43.1 vs. 41.7; p = 0.092). At the fourth week, the ODI score was significantly lower in Group A compared to Group B (p < 0.001), and this difference remained statistically significant at both the sixth and 12th weeks (p < 0.001). Both the VAS scores and ODI significantly reduced after treatment in Group A and Group B (p<0.001).

Conclusion

Ultrasound-guided caudal epidural triamcinolone acetonide injection is more effective than PT for reducing pain and improving functional disability in patients with PLID and radiculopathy. Ultrasound guidance offers a more accurate approach than PT. Further multi-center studies with larger sample sizes and long-term follow-up are needed to validate these findings.

## Introduction

Prolapsed lumbar intervertebral disc (PLID) with radiculopathy represents a significant global health concern, frequently leading to chronic low back pain, neurological deficits, and substantial disability, thereby impacting daily functional activities and quality of life [1-3]. This condition arises from the irritation or compression of lumbar nerve roots, most commonly due to disc herniation, which can manifest as pain, numbness, weakness, and altered reflexes in the distribution of the affected nerve [2,4]. The pathophysiology involves both mechanical compression and chemical irritation from inflammatory mediators released by the herniated nucleus pulposus, contributing to symptoms [2]. The economic and societal burdens associated with PLID and radiculopathy are considerable and are marked by reduced productivity and increased healthcare utilization [2,4].

Current management strategies for PLID with radiculopathy primarily emphasize conservative therapy, particularly as nonsurgical approaches are often the preferred initial management methods when "red flags" indicating severe neurological compromise are absent [2]. Physical therapy (PT) is a cornerstone of conservative management, encompassing various interventions, such as electrotherapy, traction, and lumbar stabilization exercises, which have been demonstrated to be effective in reducing low back pain and improving functional outcomes [5-7]. Therapeutic exercises and activities of daily living (ADL) instructions are crucial for neurological function recovery and long-term outcomes [7].

Interventional approaches, such as epidural steroid injections (ESIs), have been utilized for over five decades in the conservative management of PLID [8,9]. ESIs aim to deliver corticosteroids, such as triamcinolone acetonide, directly into the epidural space to reduce inflammation and pain around the affected nerve roots [9]. While fluoroscopy-guided ESIs have been the traditional standard, ultrasound-guided techniques are emerging as a viable alternative, offering advantages such as avoiding ionizing radiation and allowing real-time visualization of needle placement and injectate spread [1,10]. Caudal epidural injections are a common route, often chosen for their technical ease and lower risk profile than other epidural approaches, particularly in cases of lumbar spinal stenosis or multilevel pathology [1,11].

A synthesis of the international literature reveals the ongoing debate regarding the comparative effectiveness of different ESI approaches and PTs for lumbar disc disease [8,12]. While ESIs are widely used for symptom relief, their long-term efficacy and impact on functional recovery, especially compared with PT, remain a subject of controversy and variable outcomes [2,8,12]. Some studies suggest similar short-term effects between transforaminal and caudal steroid injections in controlling pain and improving functional disability [12]. However, inconsistencies exist, with some research indicating a potential preference for transforaminal approaches in preventing long-term recurrence of positive Lasègue tests in radiculopathy [12]. Furthermore, comparisons between ESIs and PT often highlight the immediate pain relief offered by injections versus the sustained functional improvement sought through rehabilitation [3]. This necessitates further investigation into which approach, or combination thereof, yields superior and more durable functional outcomes.

Conducting a comparative study is critically important because of the substantial patient load experiencing PLID with radiculopathy and the scarcity of local, comparative evidence [5,6,8,13]. The absence of robust local data impedes evidence-based decision-making for clinicians in resource-constrained settings [6]. Understanding the relative efficacy of ultrasound-guided caudal epidural triamcinolone acetonide injection versus PT can optimize patient care pathways, improve resource allocation, and enhance functional recovery, which is a primary focus because it directly correlates with patients' ability to perform daily activities, return to work, and improve their overall quality of life [7,14]. Therefore, the primary objective of this study was to compare the effectiveness of ultrasound-guided caudal epidural triamcinolone acetonide injection and PT in reducing pain among patients with PLID with radiculopathy over a 12-week follow-up period. The secondary objective was to compare the improvement in functional disability between the two treatment approaches using the Oswestry disability index (ODI).

## Materials and methods

This quasi-experimental study was conducted between 1st May 2022 and 31st March 2023 at the Department of Physical Medicine and Rehabilitation, Dhaka Medical College and Hospital (DMCH), Dhaka, following ethical approval from the Ethical Review Committee (ERC), DMCH (Memo no: ERC-DMC/ECC/2022/131).

The sample size was estimated to be significantly different between the ultrasound-guided caudal epidural steroid injection group and the physical therapy group on the basis of the improvement in the VAS score. The required sample size for comparing two independent means was calculated via the following formula for Cohen's d [15]:

\[ n = \frac{2 \left(Z_{\frac{1 - \alpha}{2}} + Z_{1 - \beta} \right)^2}{d^2} \]

where:

- $Z_{\frac{1 - \alpha}{2}} = 1.96$ for a two-sided 5\% level of significance (α = 0.05),

- $Z_{1 - \beta} = 0.84$ for 80\% power,

- $d = 0.7$ for a large effect size (Cohen's d).

Using these values, the calculation is as follows:

\[ n = \frac{2 \left(1.96 + 0.84 \right)^2}{0.7^2} = \frac{2 \times 7.84}{0.49} = 32 \]

Thus, the minimum required sample size was 32 participants per group, resulting in a total of 64 participants. Considering a 10% dropout rate, the final estimated sample size was increased to 71 participants.

The study was conducted with the informed written consent of 71 patients who presented with low back pain and radiculopathy but without any red flag signs. All participants had clinically diagnosed and MRI-confirmed PLIDs. Patients with structural spinal deformities such as scoliosis greater than 40°, spondylolisthesis, or infectious conditions such as Pott’s disease or inflammatory diseases such as ankylosing spondylitis were excluded from the study. Additionally, patients who had received any spinal injection in the previous month or who had undergone low back surgery, chemonucleolysis, or nucleotomy were also excluded.

The patients were non-randomly allocated into two groups: Group A (35 patients) and Group B (36 patients). Patients in Group A received a total volume of 20 ml of steroid solution consisting of triamcinolone acetonide diluted with isotonic saline via the caudal epidural approach under ultrasound guidance. A high-frequency linear ultrasound probe was used to identify the sacral hiatus, and the procedure was performed under aseptic precautions with the patient in the prone position. The average procedure time was approximately 10-15 minutes. All procedures were performed by a consultant physician specialized in Physical Medicine and Rehabilitation with more than five years of experience in ultrasound-guided interventional pain procedures. Following the procedure, patients were advised to maintain bed rest for one day and were subsequently allowed to gradually resume normal daily activities while maintaining ADL. In addition, they were instructed to perform back muscle strengthening exercises (10 repetitions, three times daily).

Patients in Group B received 12 sessions of PT conducted three times per week for four weeks. The rehabilitation program included ADL training, back muscle strengthening exercises (10 repetitions, three times daily), short-wave diathermy for 15 minutes, and intermittent pelvic traction for 15 minutes. All physiotherapy sessions were conducted by qualified physiotherapists experienced in the management of lumbar spine disorders and followed a standardized treatment protocol to ensure consistency of the intervention. Because the interventions were clearly different (injection versus physical therapy), allocation concealment and blinding of the outcome assessors were not implemented.

**Figure 1:**
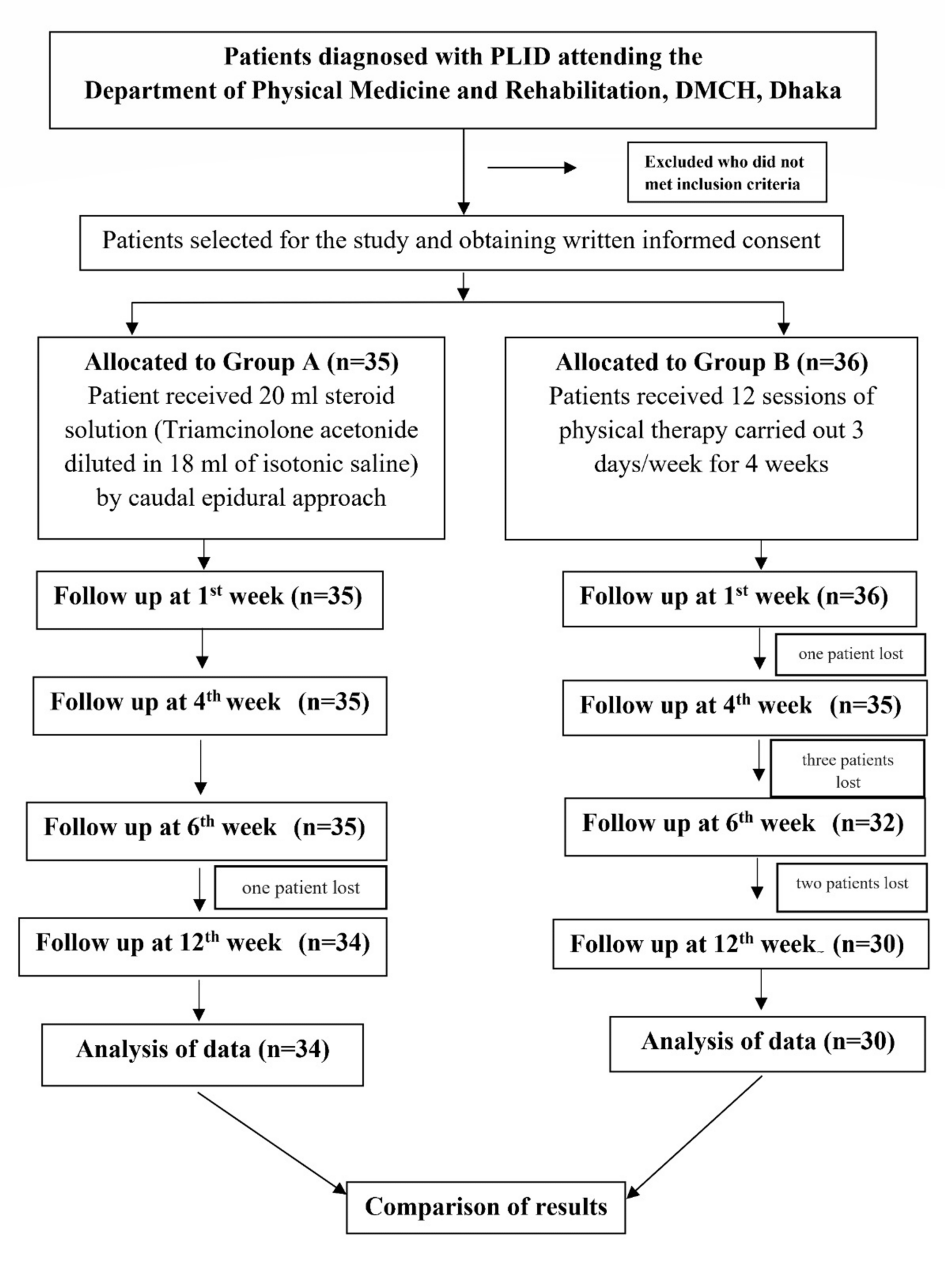
Study flowchart PLID: Prolapsed lumbar intervertebral disc

Follow-up visits were scheduled at the first, fourth, sixth, and 12th weeks. During each follow-up, pain intensity and functional disability were assessed using the visual analogue scale (VAS) and the ODI. During the follow-up period, one patient from Group A was lost in the first week and one patient from Group B in the fourth week. Additionally, three patients from each group were lost to follow-up by the sixth week. By the 12th week, a total of seven participants were lost to follow-up, resulting in a final analytical sample of 64 patients (30 in Group A and 34 in Group B). A complete-case analysis approach was applied in which participants with missing follow-up data were excluded from the final statistical analysis (Figure 1).

Pain intensity was assessed using a 10-point VAS, with 0 representing no pain and 10 indicating the worst imaginable pain. The VAS, originally described by Huskisson, is a widely used open-access tool for pain measurement [16]. The validated Bangla version of the ODI, previously applied in a Bangladeshi population by Faruk et al. [17], was used in this study to assess functional disability. Originally developed by Fairbank et al., the ODI is a widely accepted instrument for evaluating disability associated with low back pain [18]. It consists of a 10-item questionnaire that captures the patient’s perceived limitations in daily activities. The overall score ranges from 0 to 100, with higher scores indicating greater disability. Lower scores reflect the ability to perform most activities of daily living with minimal difficulty, whereas higher scores denote severe functional restriction that may lead to bed confinement [18]. Similar to the VAS, the ODI is frequently employed in clinical research to measure treatment effectiveness and monitor functional outcomes.

The statistical analysis was conducted via IBM SPSS Statistics for Windows, Version 26 (Released 2018; IBM Corp., Armonk, New York, United States). Means and standard deviations for continuous variables and frequency distributions for categorical variables were used to describe the characteristics of the total sample. Associations between categorical variables were assessed via the chi-square test and Fisher’s exact test. Group differences were evaluated by independent sample t-tests, and pre-post differences were identified via paired t tests. Here, all p-values were two-sided, and p<0.05 was considered significant.

## Results

The average age of the patients in Group A was 45.3 ± 5.1 years, and that in Group B was 44.1 ± 4.8 years. In Group A, 16 (47.1%) of the patients were female, whereas in Group B, 21 (70.0%) were female. The average body mass index (BMI) in Group A was 24.5 ± 2.6, and in Group B, it was 25.5 ± 2.8. The mean duration of symptoms was 1.1 ± 0.3 months for Group A and 1.0 ± 0.3 months for Group B. In Group A, 13 (38.2%) patients had multiple disc involvement, whereas 15 (50.0%) patients had multiple disc involvement in Group B. There were no significant differences between the groups in terms of age, sex, BMI, symptom duration, or number of discs affected (p > 0.05) (Table 1).

**Table 1:** Baseline characteristics of the patients (n=64) Group A: patients who received steroid injection via the caudal epidural approach; Group B: patients who received physical therapy

Baseline characteristics	Category	Group A (34) n (%)	Group B (30) n (%)	χ^2^/t	p-value
Gender	Male	18 (52.9)	9 (30.0)	3.45	0.064
	Female	16 (47.1)	21 (70.0)		
Age (years)	(Mean ±SD)	45.3 ±5.1	44.1 ±4.8	0.97	0.292
Occupation	Home maker	16 (47.1)	21 (70.0)	3.45	0.064
	Day laborer	6 (17.6)	2 (6.7)		
	Service holder	6 (17.6)	3 (10.0)		
	Businessman	6 (17.6)	4 (13.3)		
BMI (kg/m^2^)	(Mean ±SD)	24.5±2.6	25.5 ±2.8	-1.48	0.136
Duration of symptom (month)	(Mean ±SD)	1.1±0.3	1.0 ±0.3	1.33	0.317
Number of discs	Single	21 (61.8)	15 (50.0)	0.90	0.344
	Multiple	13 (38.2)	15 (50.0)		

At baseline, no significant difference in the VAS score was observed between the groups. However, by the fourth week, the VAS score was significantly lower in Group A than in Group B (p = 0.004). At both the sixth and 12th weeks, Group A continued to have significantly lower VAS scores than Group B (p < 0.001). Similarly, at baseline, there was no significant difference in the ODI score between the groups. By the fourth week, the ODI score was significantly lower in Group A than in Group B (p < 0.001). This trend continued at the sixth and 12th weeks, when Group A consistently presented significantly lower VAS scores than Group B (p < 0.001) (Table 2).

**Table 2:** Comparison of patients’ VAS scores and ODIs at different time points (n=64) Group A: patients who received steroid injection via the caudal epidural approach; Group B: patients who received physical therapy; VAS: visual analog scale; ODI: Oswestry Disability Index

Criteria	Group A (Mean ±SD)	Group B (Mean ±SD)	t-value	p-value
VAS				
At baseline	7.8±0.7	7.4±0.8	2.14	0.071
At the fourth week	2.6±0.9	3.6±1.6	-3.12	0.004
At the sixth week	2.5±0.9	3.8±1.5	-4.26	<0.001
At the 12th week	3.4±1.1	4.6±1.4	-3.83	<0.001
ODI				
At baseline	43.1±3.1	41.7±3.4	1.72	0.092
At the fourth week	13.1±4.9	19.3±6.1	-4.50	<0.001
At the sixth week	12.9±4.3	21.9±7.1	-6.22	<0.001
At the 12th week	18.5±4.6	25.9±7.0	-5.05	<0.001

Both the VAS score and the ODI significantly decreased after treatment in Group A and Group B (p<0.001) (Table 3).

**Table 3:** Comparison of pre-treatment and post-treatment VAS scores and the ODI in Group A and Group B Group A: patients who received steroid injection via the caudal epidural approach; Group B: patients who received physical therapy; VAS: visual analog scale; ODI: Oswestry Disability Index

Criteria	Group	Pre-treatment score	Post-treatment score	t-value (paired)	p-value
VAS	Group A	7.8±0.7	3.4±1.1	29.12	<0.001
	Group B	7.4±0.8	4.6±1.4	13.69	<0.001
ODI	Group A	43.1±3.1	18.5±4.6	43.64	<0.001
	Group B	41.7±3.4	25.9±7.0	15.30	<0.001

## Discussion

This study examined the effectiveness of ultrasound-guided caudal epidural triamcinolone acetonide injection versus PT for managing PLID with radiculopathy. Significant improvements were observed in pain and functional disability in both groups. However, the injection group showed greater short-term improvement in pain and functional disability, suggesting its superior efficacy compared to PT.

This study revealed a significant reduction in pain and functional improvement after the 12th week in both groups. Moreover, the reduction in pain and functional improvement were significantly greater in the injection group than in the PT group. Barua et al. performed a prospective study among thirty patients diagnosed with PLID and reported improvement in pain following injection [19]. A prospective study conducted by Moniruzzaman et al. [20] revealed that patients with acute and chronic low back pain due to PLID experienced significant pain reduction and functional improvement after ultrasound-guided caudal epidural corticosteroid injection. Additionally, Elashmawy et al. [21] demonstrated highly statistically significant improvements in the VAS score and ODI at one month and three months after injection compared with before injection in the ultrasound-guided caudal EPI group. Furthermore, another study by Nandi and Chowdhery reported significant differences in the VAS score and the ODI before and one month after caudal epidural corticosteroid injection and before and three months after caudal epidural corticosteroid injection [22]. A randomized controlled clinical trial by Ibrahim et al. revealed that both caudal epidural corticosteroid injection and PT significantly reduced pain on the visual analog scale after the intervention [23]. A multicenter randomized controlled trial by Arden et al. reported that, compared with a placebo, ESI reduced the VAS score and improved the ODI score. However, the efficacy of caudal epidural corticosteroid injection was transient, as no benefit was demonstrated from 6 to 52 weeks [24].

Several mechanisms have been proposed to explain the therapeutic effects of EPIs. Experimental studies suggest that radicular pain results from both mechanical compression and chemical radiculitis caused by inflammatory cytokines acting on the dorsal root ganglion. Consequently, targeted delivery of corticosteroids and local anesthetics to the affected nerve root is considered a rational treatment approach [25]. The lipophilic nature of corticosteroids enables their prolonged release from epidural fat at the injection site, leading to sustained therapeutic effects [26]. Corticosteroids act by reducing nerve edema and enhancing the expression of anti-inflammatory genes, whereas local anesthetics primarily provide immediate pain relief [27].

Ultrasound-guided caudal epidural injection offers accurate needle placement without radiation exposure, improving safety, efficacy, and patient comfort [10,28]. Although caudal epidural injection is generally considered safe, serious complications, such as dural puncture and inadvertent intravascular injection, may occur. Doo et al. [29] reported no major complications, including accidental intravascular injection, when ultrasound guidance was used during penetration of the sacrococcygeal ligament for caudal injection. In the present study, no complications were observed in any group.

PT in this study consisted of 12 sessions over four weeks, which represents a commonly used short-term rehabilitation protocol for patients with PLID and radiculopathy. However, some studies suggest that longer or more intensive rehabilitation programs may provide additional functional benefits over time. Therefore, future studies should explore whether extended PT duration could further improve long-term outcomes. The findings of the present study indicate short-term improvement in pain and functional disability during the 12-week follow-up period. However, the long-term comparative effectiveness of these interventions remains uncertain and requires further investigation.

The present study has several limitations that should be considered when interpreting the findings. First, the study employed a quasi-experimental design conducted at a single center with a relatively small sample size, which may limit the generalizability of the results. Second, although patients were followed at multiple time points, the follow-up period was limited to 12 weeks, which restricts the ability to assess the long-term sustainability of the treatment effects. Third, several potential confounding factors, such as comorbid conditions and lifestyle-related variables, were not evaluated. In addition, allocation concealment and assessor blinding were not implemented due to the nature of the interventions, which may have introduced a risk of assessment bias. Furthermore, a complete-case analysis approach was used, and participants who were lost to follow-up were excluded from the final analysis; this may have introduced potential attrition bias. The specific symptomatic disc level (e.g., L4-L5 or L5-S1) was not analyzed in this study, which may limit the interpretation of treatment response according to the level of pathology. Despite these limitations, the study provides useful preliminary evidence comparing two commonly used treatment approaches. Future multicenter studies with larger sample sizes and longer follow-up durations are recommended to confirm and extend these findings.

## Conclusions

The findings of this study suggest that ultrasound-guided caudal epidural triamcinolone acetonide injection may provide greater short-term improvement in pain and functional disability compared with PT alone among patients with PLID with radiculopathy. However, given the quasi-experimental design and relatively short follow-up period, the long-term comparative effectiveness of these treatment approaches remains uncertain. Further multicenter studies with larger sample sizes and longer follow-up durations are required to confirm these findings.
